# Treatment and Cost of Hepatocellular Carcinoma: A Population-Based Cohort Study in Taiwan

**DOI:** 10.3390/ijerph15122655

**Published:** 2018-11-26

**Authors:** Seng-Howe Nguang, Cheng-Kun Wu, Chih-Ming Liang, Wei-Chen Tai, Shih-Cheng Yang, Ming-Kun Ku, Lan-Ting Yuan, Jiunn-Wei Wang, Kuo-Lun Tseng, Tsung-Hsing Hung, Pin-I Hsu, Deng-Chyang Wu, Seng-Kee Chuah, Chien-Ning Hsu

**Affiliations:** 1 Division of Gastroenterology, Pin-Tung Christian Hospital, Pin-Tung 900, Taiwan; 01981@ptch.org.tw; 2 Division of Hepatogastroenterology, Department of Internal Medicine, Kaohsiung Chang Gung Memorial Hospital, Kaohsiung 833, Taiwan; eggclimb@cgmh.org.tw (C.-K.W.); gimy@cgmh.org.tw (C.-M.L.); wctai@cgmh.org.tw (W.-C.T.); d5637700@cgmh.org.tw (S.-C.Y.); chuahsk@cgmh.org.tw (S.-K.C.); 3 College of Medicine, Chang Gung University, Kaohsiung 833, Taiwan; 4 Division of Gastroenterology, Fu-Ying University Hospital, Kaohsiung 831, Taiwan; kumingkun1965@fy.org.tw; 5 Division of Gastroenterology, Yuan’s General Hospital, Kaohsiung 802, Taiwan; y7192@yuanhosp.com.tw; 6 Division of Gastroenterology, Department of Internal Medicine, Kaohsiung Medical University Hospital and Kaohsiung Medical University, Kaohsiung 807, Taiwan; 990326@kmu.org.tw (J.-W.W.); 1000010@kmu.edu.tw (K.-L.T.); dechwu@yahoo.com (D.-C.W.); 7 Division of Hepatogastroenterology, Department of Internal Medicine, Buddhist Tzu Chi General Hospital, Dalin Branch, Chia-Yi 622, Taiwan; dm512373@tzuchi.com.tw; 8 Division of Gastroenterology, Department of Internal Medicine, Kaohsiung Veterans General Hospital, National Yang-Ming University, Kaohsiung 813, Taiwan; williamhsup@yahoo.com.tw; 9 Department of Pharmacy, Kaohsiung Chang Gung Memorial Hospital, Kaohsiung 833, Taiwan; 10 School of Pharmacy, Kaohsiung Medical University, Kaohsiung 807, Taiwan

**Keywords:** hepatocellular carcinoma, epidemiology, liver, cost, disease burden, healthcare, Taiwan

## Abstract

Hepatitis B virus vaccination and antiviral therapies reduce the risk of hepatocellular carcinoma (HCC). However, the lifetime healthcare expenditure involved in caring for HCC patients remains unclear. We examined the use and direct costs of healthcare services for a cohort of HCC patients to the healthcare system using Taiwan national health insurance program research database between 1997 and 2012. Total medical cost for all reimbursed patient encounters, including hospitalizations and outpatient care was cumulated from HCC onset to the end of follow-up or death. The mean follow-up time was 2.7 years (standard deviation, SD = 3.3) for the entire HCC cohort. Insurance payments of approximately US$92 million were made to 5522 HCC patients, with a mean cost of US$16,711 per patient (21,350). On average, the total cost per patient per month was US$2143 (5184); it was 50% higher for advanced cirrhosis patients at the baseline but 23% lower for mild-to-moderate cirrhotic patients. In the two-part regression, patients’ underlying comorbid conditions, liver transplants, hepatectomy, and transarterial chemoembolization were associated with increased total cost, with liver transplants having the greatest impact over time. Hepatocellular carcinoma imposes substantial burden on the healthcare system. Real-world evidence on treatment and cost outcomes highlighted the needs to expand effective screening strategies and to optimize healthcare delivery to meet HCC patients’ clinical needs.

## 1. Introduction

Hepatocellular carcinoma (HCC) is the sixth most common cancer and the second leading cause of cancer-related mortality worldwide [1]. Hepatocellular carcinoma incidence varies geographically depending on the epidemiological features of cirrhosis, chronic infection from the hepatitis B virus (HBV), hepatitis C virus (HCV), alcohol hepatitis, and nonalcoholic fatty liver disease in the target populations [2,3,4]. Since the prognosis for long-term HCC outcomes is poor and is exacerbated by its negative impact on patients’ health-related quality of life, the direct and indirect costs of HCC poses a huge economic burden to society worldwide [3,5,6,7,8].

This is particularly true in Taiwan, where chronic HBV and HCV infection are prevalent as the leading causes of HCC. More than 90% of HCC patients are reactive to the hepatitis B surface antigen (HBsAg) or hepatitis C virus antibodies (anti-HCV). Although the 1984 national HBV immunization for newborns had successfully reduced HCC incidence in the vaccinated cohort over 30 years [9], for infected patients or carriers, the lifetime (30 to 78 years) risk of developing HCC was 21.7% for patients with HBV infection and 20% for HCV infected patients (30–75 years old) in Taiwan [10]. The national viral hepatitis therapy program for chronic HBV and HCV infection has been implemented and reimbursed by the National Health Insurance (NHI) program in Taiwan since October 2003 and expanded the insurance coverage for new therapies over time [11]. Data from 2008 to 2011 showed that age-adjusted HCC incidence decreased by 14% (from 2000 to 2003) and the HCC mortality decreased by 24% for 15% to 25% of infected patients [11].

Although antiviral therapies showed a reduction in both HCC morbidity and mortality in developed evidence, the magnitude of the HCC-related burden to the healthcare system remains unclear. First, only a small proportion of infected patients were eligible for the national viral therapy program. Second, the treatment of advanced liver disease and its complications should be considered while evaluating the HCC-related burden. Such understanding is critical in formulating insurance policies that prioritize healthcare services and resources to maximize disease management efficiency. To address these issues, we examine the utilization of healthcare services and the total expenditure associated with treating HCC patients. Further, we evaluate the key factors that contribute to the total cost of HCC treatment in Taiwan.

## 2. Methods

### 2.1. Data Sources

This study used the Longitudinal Health Insurance Database 2000 (LHID 2000), comprising the information of one million randomly sampled individuals who were alive in 2000 from the Registry for Beneficiaries, which includes all enrolled 23.75 million individuals [12]. The LHID 2000 comprises a highly representative sample of Taiwan’s general population because the national health insurance (NHI) program is a single-payer health insurance program that covers 99.9% of Taiwan’s population [12]. The LHID 2000 includes demographic information, diagnostics, medical treatments, prescriptions, and total costs from 1 January 1997 to 31 December 2012. The data analysts were the staff of the Center for Medical Informatics and Statistics in Kaohsiung Medical University, a site of the Application of Health and Welfare Informatics, Ministry of Health and Welfare in Taiwan. The study proposal was reviewed and approved by the Institutional Review Board and Ethics Committee of Chang Gung Medical Foundation, Taoyuan, Taiwan (IRB #201601564B1). All personal identifying information for patients was anonymous; therefore, informed consent was waived for the study.

### 2.2. Study Design and Population 

This population-based cohort study included patients 18 years of age and older who had a hospital discharge diagnosis of HCC (International Classification of Disease, 9th version (ICD-9), code 1550) in between 1997 and 2012 [7]. A cohort of newly diagnosed HCC was designed to assess the total cost of HCC. The initial date of HCC diagnosis was defined as the first recorded HCC discharge diagnosis and patients with at least 12 months of continuous enrollment in the NHI program prior to diagnosis. The cohort was followed from 365 days prior to the initial HCC diagnosis date (index date) until death, withdrawal from the NHI program, or the latest data in the dataset (censoring as of 31 December 2012). Death event was identified using discharge condition from hospitalization with fatality (coded as “4”) or with the acute terminal stage (coded as “A”) [13]. Withdrawal from the NHI program after the in-hospital death date was ascertained using the Registry for Beneficiaries.

### 2.3. Study Variables

The patients’ demographic information, comorbidities, liver diseases, and liver transplantation (LT) (ICD-9, V427) were identified in the 365 days prior to the index date. Comorbidities were assessed using the Charlson comorbidity index (CCI) [14] with the ICD-9 codes of at least 2 records from inpatient, emergency, and outpatient claims. The underlying liver diseases were: hepatitis B virus (HBV) infection (070.2, 070.3, V0261), hepatitis C virus (HCV) infection (070.41, 070.44, 070.51, 070.54, 070.70, 070.71, and V0262), other viral hepatitis (V0269), alcohol-related liver disease (ALD) (571.0, 571.3), Nonalcoholic cirrhosis (571.5), biliary cirrhosis (571.6), decompensated cirrhosis: esophageal variceal bleeding (456.0, 456.20, 530.82), ascites (789.5, 567.2,567.8, 567.9, ICD-9 procedure 54.91), hepatic encephalopathy (572.2), and others: jaundice (7824), portal hypertension (572.3), hepatorenal syndrome (5724), other sequelae of chronic liver disease (572.8). Three mutually exclusive groups: compensated cirrhosis (patients with a diagnosis of ALD, nonalcohol cirrhosis, biliary cirrhosis, but no diagnosis of decompensated cirrhosis), decompensated cirrhosis (patients with any diagnosis of decompensated cirrhosis listed above), or other chronic liver diseases (patients were not in compensate and decompensated cirrhosis groups) indicating the degree of disease severity at initial stage of HCC diagnosis.

### 2.4. Health Services Utilization and Costs

The total cost, including costs of hospitalization, length of stay, and surgeries, outpatient clinic/emergency department visits, and costs of prescription drugs for liver diseases were categorized per month for the length of each individual’s follow-up (per patient per month, PPPM). The follow-up time (survival time) began from HCC diagnosis date until either death or end of follow-up (i.e., withdrawal not due to death, or the latest date in the dataset). The costs of potentially curative treatments for HCC: hepatectomy (ICD9 procedure code, 50.22, 50.23, 50.24, 50.25, 50.26, 51.4), radiofrequency ablation (ICD9 procedure code, 50.29), and liver transplantation (NHI billing code, 75020A, 75020B, 75022A, 75022B, 75021A, 75021B), noncurative treatment for HCC: transarterial chemoembolization (TACE) (ICD-9 procedure, 88.47 plus 99.25, or 88.47 plus 38.86, or 88.47 plus 99.29), and rescue therapy: transjugular intrahepatic portosystemic shunt (TIPS) procedure (ICD9 procedure 39.1) were categorized and analyzed their impacts on the total cost of HCC [15]. Transarterial chemoembolization for liver cancer has been proven to be useful in local tumor control, to prevent tumor progression, prolong patients’ life for those who are not candidates for surgery [16]. The TIPS procedure is one of complex procedures performed for esophageal variceal bleeding, refractory ascites and portal hypertension in patients with HCC [17]. Sorafenib was not available in Taiwan until late 2012, which was not in the time frame of analysis (1997–2012). The prescriptions for liver diseases were interferons/ribavirin, nucleos(t) ide analogues (NUCs), diuretics, propranolol, and liver protectants. Costs were viewed from the perspective of the payer. The costs for all claims in the follow-up months were adjusted for each year’s inflation using the 2012 average consumer price index in New Taiwan dollars (NT$) [18]. The exchange rate for NT$ to US$ was 31.5 for 2016 in this study.

### 2.5. Statistical Analysis

Patient characteristics, healthcare service utilization, and cost estimates were summarized as counts with proportions for categorical data and as the mean (standard deviation, SD) and median (interquartile range, IQR: 25th to 75th percentile) for continuous data. The categorical variables between groups were compared using the χ^2^ test. The Cox proportional hazards regression model was used for factors associated with follow-up mortality. Similarly, factors that impacted total cost per person were assessed using a two-part model with multiple linear regressions after logarithmic transformation of the cost [19]. The statistical significance level was 5% and a two-sided *p* value < 0.05 was considered significant. All statistical analyses were performed using SAS software version 9.4 (SAS Institute Inc., Cary, NA, USA).

## 3. Results

### 3.1. Patient Characteristics

Table 1 presents the characteristics of 5522 patients, newly diagnosed with HCC, during the period 1997–2012. Male predominance (69.18%) was observed among the sample. The mean age was 64 (±13.58) years old and 48.61% patients were aged >65 years old at HCC diagnosis. More than 50% of the HCC cohort had viral hepatitis (56%), including HBV (27.74%), HCV (22.46%), and a combination of HBV or HCV infection (6.28%). Among the study cohort, 62% had pre-existing liver cirrhosis; approximately 25% had cirrhosis-related complications: 10.47% of patients had ascites; 11.21% experienced gastroesophageal variceal bleeding, and 5.67% had hepatic encephalopathy; and 12 patients (0.22%) had prior liver transplants.

### 3.2. Survival Outcomes

**Figure 1:**
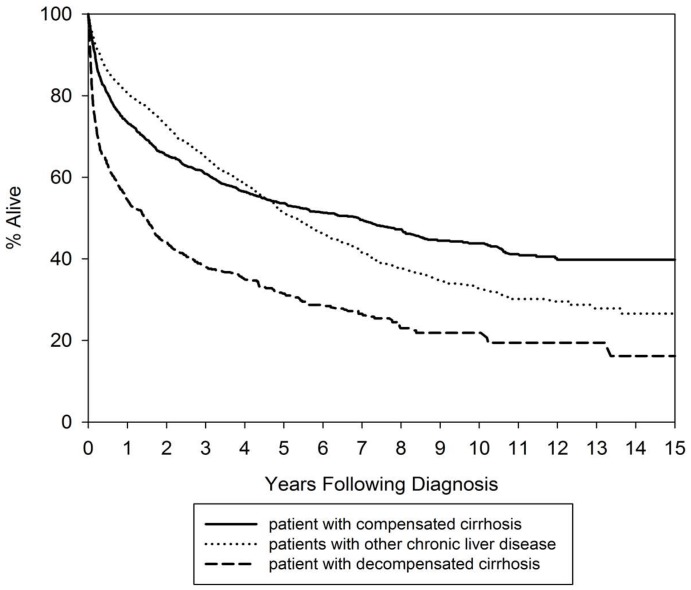
Kaplan-Meier survival analysis by disease stage. The log rank test for the group of compensated cirrhosis vs. the group of other chronic liver disease, *p* = 0.906; for the group decompensated cirrhosis vs. the group with other chronic liver disease, *p* < 0.001.

The overall mortality rate due to any cause is 44% (2432/5522). The overall 1, 3 5 year survival was 71.68%, 57.14% and 47.82%, respectively. Table 2 presents the all-cause mortality rate was higher (54.2%) for patients with decompensated cirrhosis than those with compensated cirrhosis (40.7%) and other liver diseases (40.5%). Figure 1 presents Kaplan-Meier survival curve for the HCC patients by stage (a long-rank test, *p* < 0.0001).

### 3.3. Health Service Utilization

Table 2 shows that 76.6% of the patient cohort received a prescription for liver disease. Around 11.9% received nucleoside/nucleotide treatment for HBV infection; 2.6% patients received interferon-based antiviral for the treatment of HCV infection; half of the patients received liver protectants and diuretics; and 26.1% received propranolol for the control of portal hypertension and gastro-esophageal varices. Additionally, 106 liver transplant surgeries were performed in 61 patients and 6106 liver resections and procedures were performed in 2326 patients (42.1%) following HCC diagnosis. In general, the use rate of surgical interventions was lower in patients with decompensated cirrhosis than those with less advanced liver disease. Although sample size is small, liver transplantation and TIPS were slightly higher in patients with decompensated cirrhosis than those with compensated cirrhosis.

### 3.4. Total Cost for Caring for Hepatocellular Carcinoma Patients

Over the study period, the total healthcare expenditure (insurance payments) for treating HCC patients was approximately US$92 million (92,269,551), including US$53.4 million (58%) for hospital care and US$38.7 million (42%) for outpatient and emergency department services (Table 3). The total payment was approximately US$44 million (48%) for TACE, US$21 million (23%) for hepatectomy and US$6.3 million (6.8%) for liver transplantations.

**Figure 2:**
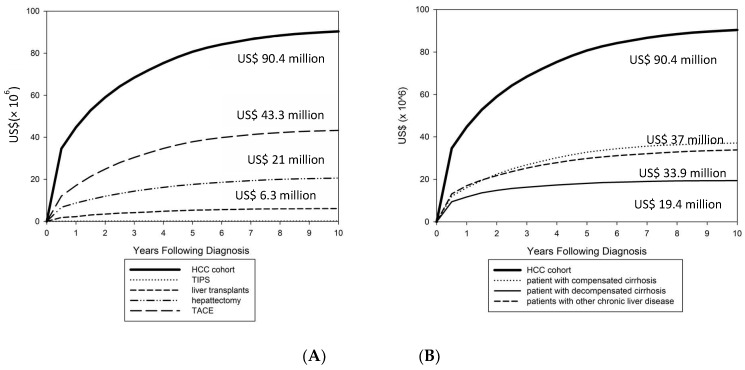
The 10-year cumulative costs for HCC patients by surgical intervention (**A**) and by stage of liver disease (**B**). US$90.4 million (90,377,380) for the entire HCC cohort; US$43.3 million (43,296,479) for patients received TACE procedures; US$21million (20,600,273) for hepatectomy; US$158,068 for TIPS; and US$6.3 million (6,294,255) for liver transplantation; US$37 million (37,102,854) for compensated cirrhosis; US$33.9 million (33,862,713) for other liver disease; US$19.4 million (19,411,813) for with decompensated cirrhosis.

Figure 2A shows that surgical interventions and liver transplantation contributed to a significant proportion of the total 10-year cumulative expenditure. Surgical interventions (most were hepatectomy and TACE) expenditure changes (increase) were higher in the first three years of follow-up (from 47% to 56%) and slow and sustained afterward (from 58% to 60%) in the 10-year period. The trend changes in liver transplantation expenditure revealed the same pattern (from 5.3% to 6.1%, then 6.2% to 6.8%) in the 10-year period.

Table 4 shows that the mean follow-up time (survival time) was approximately 2.7 (±3.3) years (median, 1.4 years; IQR, 0.3–3.7) for the entire HCC cohort. Each patient experienced an average of 4 hospitalizations and there were 81 outpatient visits during the study period. The mean length of hospital stay was 10 (±12.5) days. The aggregated mean total cost per patient was US$16,711 (±21,350), including US$9721 (±11,811) for inpatient care and US$6989 (±14,726) for outpatient visits. The PPPM cost was US$2143 (±5184), with hospitalizations accounting for 83.7% of this cost.

Figure 2B shows the estimated cumulative health care expenditure over 10 years for the HCC cohort overall and by disease stage. Because of the shorter survival time of HCC patients with decompensated cirrhosis, the overall 10-year cumulative costs were lower (US$19.4 million) compared with those with compensated cirrhosis (US$37.1 million) and with other liver diseases (US$33.9 million). Shorter-term survivors accumulated cost at a faster rate in early period of observed survival time (i.e., 6 months), suggesting intensive resource use; and long-term costs sustained a slow rate afterward.

For monthly resource use, Table 4 shows that overall PPPM was higher (US$3212 ± 5002) for patients with decompensated cirrhosis than those who presented chronic liver disease (US$1913 ± 5658) and compensated cirrhosis (US$1643 ± 4652) at the baseline. For most resource use categories, hospitalization costs dominated the total cost per month for each HCC patient, with the highest PPPM (88%) for patients with decompensated cirrhosis, and the PPPM was 82% and 80% for patients with other chronic liver diseases and compensated cirrhosis, respectively.

Table 5 presents the factors associated with higher total lifetime cost per person for HCC care. Liver transplantation (β = 1.47 ± 0.06), hepatectomy (β = 0.15 ± 0.06), TACE (β = 0.44 ± 0.05), the complex of baseline comorbid conditions (β = 0.07 ± 0.01), and follow-up length (β = 0.006 ± 0.0004) were associated with higher expenditure (all *p* < 0.01); in contrast, an age of over >65 years (β = −0.3 ± 0.11) was significantly associated with lower health expenditure (*p* = 0.005).

## 4. Discussion

The study results demonstrate all the health resource components used for treating HCC patients over the course of the disease at the population level in the Taiwan healthcare system. The all-cause mortality rate was high (42%) in the HCC cohort with a mean follow-up time of approximately 3 years. We found that mean total direct cost per HCC patient was US$16,711 ± 21,350 (NT$526,382 ± 672,510), which is close to the previous lifetime cost estimate (NT$511,563) in 2002 [5]. Adjusting for the follow-up length, the PPPM cost was US$2143 ± 5184 (approximately NT$67,509).

According to the estimated HCC incidence rate of HCC of 30 cases per 100,000 in Taiwan’s general population (23,000,000) [20], the annual insurance payment for HCC patient (*n* = 6900) care is estimated to be US$1.8 billion (approximately NT$5.6 billion, with an exchange rate of 31.5 in 2016).

In actual clinical practice, the PPPM cost was high among patients with decompensated cirrhosis, resulting from a high hospitalization rate and high cost of surgical interventions (liver transplantation, TIPS). Further, the overall survival rate was lowest for these patients compared to patients with mild-to-moderate chronic liver disease. These findings were consistent with previous studies showing that medical costs for treating HCC patients with advanced liver disease at diagnosis were higher than other etiology of liver diseases in the western population [7,8,21]. Among patients with compensated cirrhosis (86.8%), the mean cost (US$680 ± 1527) for liver disease prescriptions was slightly higher (1.5 to 1.6 times) than for other HCC patients, while liver transplantation (US$5310 ± 17,725) was two to three times higher in than for other HCC patients.

Compared to the cost analysis at the population level, the cumulative treatment cost for HCC rapidly increases during the initial two years of follow-up for patients with a short survival time in the present study, similar to the US Medicare population (survival time, mean 12.3±18.1 months) in 2009 in US dollar [22]. The cost of HCC care in the present study and other populations confirmed that PPPM cost varies with the initial stages of HCC and lifetime cost depends on the use of resources during the survival time [7,22].

Hepatocellular carcinoma is highly fatal and is the second most common cancer that causes mortality in Taiwan [23]. This study provides a comprehensive view of the cost burden of HCC care in Taiwan’s healthcare setting. Our findings have several important implications for HCC treatment decisions and healthcare policies from a third-party payer perspective. First, although HBV- and HCV-related HCC incidence has declined between 2004 and 2011, this decline is not enough to slow down the increased need for healthcare resources at the population level. For instance, the 2003 national viral hepatitis program only covered 15 to 25% of infected patients in the Taiwanese population [11]. A majority of patients aged over 50 years remained asymptomatic or were not eligible for the reimbursed viral therapy program. Further research is warranted to investigate the barriers to accessing reimbursed antiviral therapies in practice.

Estimating costs for HCC treatment without considering patients’ comorbid conditions, apart from liver-related symptoms or medications, would result in underestimating the economic burden of HCC. In the present study, a total cost accounting approach for patient comorbidity would help us to further identify whether and which comorbid conditions form a barrier to comprehensive HCC treatment. For instance, to optimize HCC treatment, a multidisciplinary team approach has been suggested to provide an individualized treatment plan, covering comorbid illnesses, the functional status of patients, and treatment options ranging from curative surgery for patients with early-stage HCC to palliative or hospice care for patients with metastatic HCC [24].

Over the past decade, several types of risk screening for early HCC detection [25,26], newer and more expensive direct-acting anti-HCV therapies [27], and HCC treatment options including surgery (e.g., living donor liver transplantation) and procedures (e.g., TACE, chemotherapy with sorafenib) [28,29] have been introduced in the healthcare system. Although innovative treatments and technologies increases the probability of better treatment outcomes, they lead to rising costs and increased spending for the healthcare system. However, while the PPPM cost for HCC patients with severe liver disease at diagnosis were higher than for those with mild to moderate liver disease, the survival time was shorter, indicating that substantial resources were allocated to patients with small health gains. This provides important insights for developing optimal resource allocations strategies regarding risk screening for the general population or reducing the risk for advanced liver disease and/or HCC in targeted patients with mild liver disease caused by either HBV or HCV infection. Further research is required to evaluate the cost-effectiveness of different intervention types for society.

This study was subject to certain limitations common to studies using claims data. First, laboratory results regarding HCC tumor size of tumor or severity are not available in the NHI dataset, which is a common drawback of all claims databases. This limitation was addressed by using the proxy of liver disease diagnosis (chronic liver disease, compensated cirrhosis, or decompensated cirrhosis) at baseline. Because the degree of cirrhosis complications at the time of HCC initial diagnosis are associated with treatment modality, survival time, resource utilization, and total cost [15,30], although treatment choices can vary depending on accessibility of treatment and regions [31]. Second, the proportion of HBV infection in the present study (27.74%) was lower than the 56.02% (2005–2011) for samples in the multicenter setting [18], which indicates potential coding errors or incomplete data, or the proportion of HBV in the LHID 2000 is not representative of the general population, although the demographics of the 1 million random samples are representative of the Taiwanese population.

## 5. Conclusions

The current study highlights the clinical burden of HCC to the healthcare system is significant. The mortality rate was over 50% among patients with decompensated cirrhosis at the time of HCC diagnosis, whose median survival was 6 months in the study. Although cumulative life time costs for treating HCC was lower than those with less advanced liver disease, PPPM costs were higher for a group of HCC patients with decompensated cirrhosis. This study’s findings representing real-world cost estimates of HCC care provide insightful information for future cost-effectiveness analysis of early interventions (i.e., screening and novel therapies), resource allocation, and health insurance reimbursement policies to improve the health outcomes of HCC patients.

## Figures and Tables

**Table 1:** Baseline characteristics of HCC patients.

	Overall	
	*n*	%
Gender		
Male	3820	69.18%
Female	1702	30.82%
Age at HCC diagnosis, years (mean ± SD)	63.81 ± 13.58	
Age group, years		
<45	536	9.71%
45–55	964	17.46%
55–65	1338	24.23%
>65	2684	48.61%
CCI score (mean ± SD) (excluding liver-related diseases)	1.79 ± 0.78	
0	1486	26.91%
1	1619	29.32%
2	810	14.67%
≥3	1607	29.10%
Prior liver disease		
None	2316	41.94%
HBV only	1532	27.74%
HCV only	1240	22.46%
ALD only	87	1.58%
HBV + HCV (or ≥2 items liver diseases *)	347	6.28%
Prior complications		
Nonalcoholic cirrhosis	3020	54.69%
Biliary cirrhosis	23	0.42%
Alcoholic cirrhosis	385	6.97%
Ascites	578	10.47%
Variceal bleeding	619	11.21%
Hepatic encephalopathy	313	5.67%
Other decompensated cirrhosis	326	5.90%
Prior liver transplantation	12	0.22%

≥2 items liver diseases *: (other than HBV + HCV, e.g., HBV or HCV + ALD, HBV or HCV + other viral hepatitis). Hepatocellular carcinoma (HCC); standard deviation (SD); Charlson Comorbid Index (CCI); Hepatitis B virus (HBV); Hepatitis C (HCV); Alcohol liver disease (ALD)

**Table 2:** Health services use and mortality rate among HCC patients during the study period, by disease stage.

	Overall (*n* = 5522)		Compensated Cirrhosis (*n* = 2007)		Decompensated Cirrhosis (*n* = 1392)		Others (*n* = 2122)	
Liver transplantation	61	1.1%	30	1.5%	23	1.7%	8	0.4%
Hepatecotomy	902	16.3%	372	18.5%	87	6.2%	443	20.9%
TACE	1737	31.5%	862	42.9%	295	21.2%	580	27.3%
TIPS	6	0.1%	2	0.1%	4	0.3%	0	0
Prescriptions drugs	4230	76.6%	1743	86.8%	1033	74.2%	1454	68.5%
NUC	659	11.9%	320	15.9%	157	11.3%	182	8.6%
INFs/RBV	143	2.6%	77	3.8%	23	1.7%	43	2.0%
Diuretics	2802	50.7%	1159	57.7%	860	61.7%	783	36.9%
Propranolol	1443	26.1%	595	29.6%	412	29.6%	436	20.5%
Liver protectants	3214	58.2%	1376	68.6%	773	55.5%	1065	50.2%
All-cause mortality	2432	44.0%	817	40.7%	755	54.2%	860	40.5%

NUC = nucleoside/nucleotide; INFs = interferons; RBV = ribavirin; Others: other chronic liver disease. TACE = transarterial chemoembolization; TIPS = transjugular intrahepatic portosystemic shunt. Overall 106 liver transplantation surgeries and 6106 liver resections and procedures were performed in 42.1% HCC patients (*n* = 2326).

**Table 3:** Heath service use and costs among HCC patients.

	Patient		Health Services and Costs				
	*n*	%	Mean	SD	Median	25th	75th
Follow-up, years	5522		2.7	3.3	1.4	0.3	3.7
Total cost							
US$ per person			16,711	21,350	10,512	4800	21,093
US$ PPPM			2143	5184	797	323	2177
Hospitalization	5522						
Number of hospitalization per person			4.3	4.4	3	1	6
LOS, days			10.0	12.5	7	3	12
US$ per person			9721	11,811	5946	2533	12,544
US$ PPPM			1793	4560	504	148	1706
Outpatient/emergency department visit	5522						
Number of visit per person			81.4	115.1	38	8	110
US$ per person			6989	14,726	2922	666	8063
US$ PPPM			349	1417	166	87	305
Prescription for liver disease (US$ per person)	4230	76.6%	533	1389	49	7	285
NUC	659	11.9%	1818	2206	943	182	2742
INFs/RBV	143	2.6%	3867	2364	3735	2548	4715
Diuretics	2802	50.7%	23	58	5	1	21
Propranolol	1443	26.1%	17	1120	74	0	488
Liver protectants	3214	58.2%	129	230	34	7	139
Surgery or procedure (US$ per person)							
Liver transplantation	61	1.1%	3908	12,474	1743	1152	2800
Hepatecotomy	902	16.3%	1007	3186	480	251	953
TACE	1737	31.5%	1273	2998	716	385	1337
TIPS	6	0.1%	2111	1085	1664	1307	3210

Total cost = sum of costs for health service use in hospitalization and outpatient visits; PPPM = per person per month; 1 US$ = 31.5 NT$ in 2016 exchange rate; NUC = nucleoside/nucleotide; INFs = interferons; RBV = ribavirin; Surgery: portosystemic shunt; TACE = transarterial chemoembolization; TIPS = transjugular intrahepatic portosystemic shunt. The total direct medical cost for treating HCC, US$92 million, including US$53.4 million for hospital care (58%), and US38.7 million (42%) for outpatient and emergency department services.

**Table 4:** Heath service use and costs among HCC patients, by disease stage.

	Compensated Cirrhosis					Decompensated Cirrhosis					Other Chronic Liver Diseases				
	Mean	SD	Median	25th	75th	Mean	SD	Median	25th	75th	Mean	SD	Median	25th	75th
Follow-up, years	3.0	3.1	2.0	0.6	4.4	1.6	2.4	0.5	0.1	1.8	3.2	3.8	1.5	0.3	4.7
Total cost															
US$ per person	18,892	20,852	12,998	6350	24,284	13,992	20,888	7646	3269	16,865	16,431	21,896	10,230	5004	19,899
US$ PPPM	1643	4652	654	310	1465	3212	5002	1626	557	3881	1913	5658	689	253	1923
Hospitalization															
Number of hospitalization per person	5.1	4.7	4	2	7	3.6	3.9	2	1	4	4	4.4	2	1	5
LOS, days	9.2	11.7	6	3	11	11.4	13.2	7	4	14	10.2	12.9	7	3	13
US$ per person	10,878	12,258	7035	3088	14,400	9378	13,146	5039	2188	11,378	8852	10,270	5562	2343	11,468
US$ PPPM	1320	4444	380	139	1015	2824	4461	1132	316	3512	1565	4628	409	98	1399
Outpatient/emergency department visit															
Number of visit per person	95.1	115.5	59	18	131	45.7	75.2	12	20	57	91.8	130.5	41	11	121
US$ per person	8014	13,502	4393	1304	9800	4614	11,464	1310	211	4916	7579	17,342	2893	795	8354
US$ PPPM	323	730	174	105	294	388	1194	179	79	355	348	1946	151	75	281

Total cost = sum of costs for health service use in hospitalization and outpatient visits; PPPM = per person per month; 1 US$ = 31.5 NT$ in 2016 exchange rate; LOS = length of stay.

**Table 5:** Factors associated total lifetime cost per patient.

Variable	Coefficient	95% CI		*p* Value
Intercept	9.10	8.9215	9.2776	<0.0001
Male vs. female	0.898	−0.0221	0.2016	0.1156
45–55 vs. ≤45 years	0.0418	−0.0721	0.1557	0.4716
55–65 vs. ≤45 years	−0.0515	−0.1873	0.0843	0.4575
>65 vs. ≤45 years	−0.3015	−0.5133	−0.0896	0.0053
Compensated cirrhosis *	−0.016	−0.1276	0.0957	0.7792
Decompensated cirrhosis *	0.0982	−0.0351	0.2315	0.1487
INFs/RBV vs. none	−0.06	−0.2672	0.1472	0.5701
Liver transplantation	1.4699	1.348	1.5918	<0.0001
Hepatectomy	0.1526	0.0422	0.2629	0.007
TACE	0.4448	0.3434	0.5462	<0.0001
TIPS	0.4796	−0.6079	1.5672	0.3874
CCI score	0.0745	0.0451	0.1039	<0.0001
Length of follow-up	0.0064	0.0056	0.0073	<0.0001

* reference group = Prior other chronic liver diseases; INFs = interferons; RBV = ribavirin; TACE = transarterial chemoembolization; TIPS = transjugular intrahepatic portosystemic shunt; CCI = Charlson comorbidity index.
